# Absence of in vivo selection for K13 mutations after artemether–lumefantrine treatment in Uganda

**DOI:** 10.1186/s12936-016-1663-1

**Published:** 2017-01-09

**Authors:** Betty Balikagala, Toshihiro Mita, Mie Ikeda, Miki Sakurai, Shouki Yatsushiro, Nobuyuki Takahashi, Shin-Ichiro Tachibana, Mary Auma, Edward H. Ntege, Daisuke Ito, Eizo Takashima, Nirianne Marie Q. Palacpac, Thomas G. Egwang, Joseph Okello Onen, Masatoshi Kataoka, Eisaku Kimura, Toshihiro Horii, Takafumi Tsuboi

**Affiliations:** 1 Division of Malaria Research, Proteo-Science Center, Ehime University, 3 Bunkyo-cho, Matsuyama, Ehime 790-8577 Japan; 2 Department of Molecular and Cellular Parasitology, School of Medicine, Juntendo University, Tokyo, 113-8421 Japan; 3 Department of International Affairs and Tropical Medicine, School of Medicine, Tokyo Women’s Medical University, Tokyo, Japan; 4 Health Research Institute, National Institute of Advanced Industrial Science and Technology (AIST), Takamatsu, Japan; 5 St. Mary’s Hospital LACOR, Gulu, Uganda; 6 Department of Molecular Protozoology, Research Institute for Microbial Diseases, Osaka University, Suita, Japan; 7 Med Biotech Laboratories, Kampala, Uganda; 8 Department of Biology, Faculty of Science, Gulu University, Gulu, Uganda

**Keywords:** In vivo selection, *pfkelch13*, Artemether–lumefantrine, Drug resistance, Polymorphisms, *Plasmodium falciparum*

## Abstract

**Background:**

Individual drug treatment may select resistant parasites in the human body, a process termed in vivo selection. Some single nucleotide polymorphisms in *Plasmodium falciparum* chloroquine-resistance transporter (*pfcrt*) and multidrug resistance gene 1 (*pfmdr1*) genes have been reportedly selected after artemether–lumefantrine treatment. However, there is a paucity of data regarding in vivo selection of *P. falciparum* Kelch propeller domain (*pfkelch13*) polymorphisms, responsible for artemisinin-resistance in Asia, and six putative background mutations for artemisinin resistance; D193Y in *ferredoxin*, T484I in *multiple resistance protein 2*, V127M in *apicoplast ribosomal protein S10*, I356T in *pfcrt*, V1157L in *protein phosphatase* and C1484F in *phosphoinositide*-*binding protein*.

**Methods:**

Artemether–lumefantrine efficacy study with a follow-up period of 28 days was conducted in northern Uganda in 2014. The above-mentioned genotypes were comparatively analysed before drug administration and on days; 3, 7, and 28 days after treatment.

**Results:**

In 61 individuals with successful follow-up, artemether–lumefantrine treatment regimen was very effective with PCR adjusted efficacy of 95.2%. Among 146 isolates obtained before treatment, wild-type alleles were observed in 98.6% of isolates in *pfkelch13* and in all isolates in the six putative background genes except I356T in *pfcrt*, which had 2.4% of isolates as mixed infections. In vivo selection study revealed that all isolates detected in the follow-up period harboured wild type alleles in *pfkelch13* and the six background genes.

**Conclusion:**

Mutations in *pfkelch13* and the six background genes may not play an important role in the in vivo selection after artemether–lumefantrine treatment in Uganda. Different mechanisms might rather be associated with the existence of parasites after treatment.

**Electronic supplementary material:**

The online version of this article (doi:10.1186/s12936-016-1663-1) contains supplementary material, which is available to authorized users.

## Background

Since the mid-2000s, artemisinin-based combination therapy (ACT) has been deployed as first-line treatment for uncomplicated *Plasmodium falciparum* malaria in nearly all malaria endemic countries [1]. The wide-scale deployment has been regarded as one of the central causes of the recent decline in malaria related morbidity and mortality rates [2]. However, since the first report of artemisinin-resistant *P. falciparum* malaria in Western Cambodia [3], geographical areas of artemisinin resistance have steadily spread into the Greater Mekong sub-region [4, 5]. In Africa, although previous clinical trials have demonstrated rapid parasite-clearance after ACT treatment [6], there is a global concern that artemisinin resistance may invade this region following the path previously observed in chloroquine and sulfadoxine/pyrimethamine resistance [7–10].

In 2014, *pfkelch13* (PF3D7_1343700) was identified as a useful molecular marker for tracking the emergence and spread of artemisinin resistant *P. falciparum*. *Pfkelch13* encodes a 726 amino acid protein with a broad-complex, tramtrack, bric-a-brac/poxvirus and zincfinger (BTB/POZ) domain and a C-terminal 6-blade propeller domain [11]. Some mutations in these two domains are associated with delayed parasite-clearance time following artemisinin treatment in Southeast Asia [4, 11]. In addition, a recent genome-wide association study has identified several single nucleotide polymorphisms (SNPs) that are assumed to be background genetic changes for artemisinin resistance, these include; D193Y in *ferredoxin* (*fd*), T484I in *multidrug resistance protein 2*+ (*mdr2*), V127M in the *apicoplast ribosomal protein S10* (*arps10*), I356T in *chloroquine*-*resistance transporter* (*crt*), V1157L in *protein phosphatase* (*pph*) and C1484F in *phosphoinositide*-*binding protein* (*pibp*) [12]. In Africa, polymorphisms in these genes have been occasionally observed, but mostly different from those reported in Southeast Asia [13, 14]. Therefore, it remains unclear whether the existence of these mutations in Africa is a consequence of selection induced by anti-malarial usage.

During the period of antimalarial treatment, less susceptible parasites can be selected in the human body, a process termed in vivo selection. This is because the treatment would create drug concentration circumstances that are sufficient to kill susceptible, but not less susceptible parasites. Previous investigations revealed that artemether–lumefantrine (AL) treatment selected for parasites harbouring alleles with K76 in *pfcrt* and N86, 184F and D1246 in *pfmdr1* [15–17]. However, the possibility of similar in vivo selection has not been fully investigated in *pfkelch13* [18] and the putative background genes.

In Uganda, AL was adopted as a first line treatment for uncomplicated malaria in 2004, but actual implementation was in 2006. So far, marked clinical efficacy has been reported to this regimen; 0–0.5% of cases with residual parasites by day 3 [19–28] and 1.0–6.0% of recrudescence [20, 21, 24, 26]. All these values were determined microscopically. Molecular assessments using high-sensitive PCR are able to detect sub-microscopic infection of parasites during follow-up after anti-malarial treatment, some of which might possess resistant phenotypes [29]. In this study, AL clinical efficacy in Gulu, Northern Uganda was assessed. Recruited individuals were followed up for 28 days after treatment and presence of parasites was determined by polymerase chain reaction (PCR) to detect sub-microscopic infections. Genotyping of *pfkelch13* polymorphisms, and putative background SNPs for artemisinin resistance in *P. falciparum* recurrent infections was done to evaluate whether AL treatment selected for polymorphisms in a region where it has been used for a long time.

## Methods

### Study site and patients

The study was conducted at the peak of malaria transmission between May–July and October–November 2014 at St Mary’s Hospital Lacor in Gulu district, Northern Uganda. Malaria transmission in the study region is perennial with an estimated prevalence of >60%, and entomological inoculation rate (EIR) of 100 or more infective mosquito bites per person per year [30]. *Anopheles funestus* is the major mosquito vector and a few infections are due to *Anopheles gambiae* [31]. Malaria control measures in the region include, indoor residual spraying (IRS), long-lasting insecticide-treated nets (LLINs), malaria case management with ACT and intermittent preventive treatment during pregnancy (IPTp). In particular, IRS has been scaled-up since 2009 to cover 10 high-malaria burden districts (Apac, Kole, Gulu, Amuru, Nwoya, Pader, Agago, Kitgum, Oyam and Lamwo) in the mid-northern region [32]. Symptomatic individuals with *P. falciparum* positive results by rapid diagnostic test (RDT) were referred to the study physicians. The criteria for recruitment are shown in Additional file 1. Individuals who had received anti-malarial treatment within two weeks prior to enrollment were excluded from AL efficacy study but recruited in the in vivo selection study.

### Ethics, consent and permissions

Before enrollment, written informed consent was obtained from the participants’ parents or guardians, and children aged ≥7 years were assented. The study was reviewed and approved by Lacor Hospital Institutional Research and Ethics Committee (LHIREC) (Study protocol number LHIREC 008/05/2013 and 021/09/13) and regulatory approval was obtained from the Uganda National Council for Science and Technology (UNCST) (HS 1395).

### Artemether–lumefantrine treatment and follow-up assessment

For the efficacy study, AL (Coartem^®^, Novartis 20 mg artemether/120 mg lumefantrine tablets) was orally administered twice daily for 3 days and follow-up assessments were performed on days 1, 2, 3, 7, and 28 after initial drug treatment. Dosage of oral Coartem^®^ was adjusted according to the participant’s body weight: one (5–14 kg), two (15–24 kg), or three (25–34 kg) tablets. The drug was given as directly observed treatment (DOTS) for all patients by study nurses and physicians. After each treatment, patients were carefully observed for 30 min, and the same dose was re-administered if vomiting occurred. Rescue treatment regimen (dihydroartemisinin–piperaquine) was administered daily for 3 days to any individuals who failed on the initial AL therapy. If the recruited patients developed severe malaria during follow-up, they were referred to the hospital for parenteral artesunate.

At enrollment, venous blood samples (1 mL) were obtained from the cubital vein before initial treatment except for children <2 years where finger prick sampling was performed. A finger-prick blood sample of 100 μL was obtained at each follow-up visit. Blood was spotted on chromatography filter paper (ET31CHR; Whatman Limited, Kent, UK). Haemoglobin (Hb) concentration was measured using a portable spectrophotometer Hemocue Hb 201 (HemoCue, Ängelholm, Sweden) on days 0 and 28 or on the day of late clinical failure. Anaemic patients with Hb level <10.0 g/dL were treated with Ferrous sulphate tablets for 14 days. Plasma concentrations of artemether and lumefantrine were not measured. Treatment outcomes were classified according to WHO guidelines for areas of intense malaria transmission as: adequate clinical and parasitological response (ACPR), early treatment failure (ETF), late clinical failure (LCF) and late parasitological failure (LPF) [33].

### Microscopic and molecular diagnosis of malaria parasites

Thick and thin blood smears were stained with 2% Giemsa for 30 min. The number of parasites was counted per 200 white blood cells (WBCs), assuming 8000 WBC/µL. Parasite density was calculated by averaging independent counts made by two microscopists. Discordant results (difference in parasite density of >50%) were re-examined by a third microscopist and, parasite density calculated by averaging two closest counts. Slides were considered negative if after examination of thick smears, no parasite was detected in 100 high power fields. Parasite DNA was extracted from a quarter of dried blood spot (25 µL) using a QIAamp DNA Kit (Qiagen, Hilden, Germany) [34]. In all first visit and follow-up cases, *P. falciparum* infections were assessed by species-specific PCR as previously described [35]. Genotyping of *merozoite surface protein 1* (*msp1*), *merozoite surface protein 2* (*msp*-*2*) and *glutamate rich protein* (*glurp*), was also performed to differentiate between recrudescence and new infections on day 0 and the day of positive infection [36, 37]. Nested PCR products were analysed by electrophoresis using 2% agarose for *msp*-*1* and *msp*-*2* and 1.5% agarose for *glurp*. Patient samples were run side by side. Gel images were digitised and molecular weights determined using imagej software [38], an open platform for scientific image analysis. Densitometric curves were generated for each gel lane, and dominant bands in each lane were assigned molecular weights. Using reference strains, alleles were considered the same if molecular weights were within 10 bp for *msp 2* and 20 bp for *glurp*.

### Genotyping of drug-resistant genes

Polymorphisms in *pfcrt* (K76T) and *pfmdr1* (N86Y, Y184F, S1034C, N1042D and D1246Y) were determined as reported [39, 40]. K13-propeller domain was amplified by nested PCR, covering almost all the six propeller domain sequences, as described [11]. The sequences were aligned using MUSCLE in MEGA software, version 6.06 [41] with *P. falciparum* 3D7 full-length sequence of K13-propeller domain (PF3D7_1343700) from PlasmoDB [42] as reference.

Six background mutations for artemisinin resistance (D193Y in *fd*, T484I in *mdr2*, V127M in *arps10*, I356T in *crt*, V1157L in *pph* and C1484F in *pibp*) were amplified by multiplex PCR using gene specific primers (Additional file 2). In brief, 10 μL reaction mixture consisted of 1 μL of DNA template, 0.5 μM of 6-primer sets, and PrimeSTAR Max DNA Polymerase (Takara Bio Inc., Otsu, Japan). Cycling conditions were: denaturation at 98 °C for 10 s, followed by 40 cycles of amplification (98 °C for 10 s, 60 °C for 10 s, and 72 °C for 90 s), with a final elongation period of 90 s at 72 °C. ExoSAP-IT Kit was used for the purification of PCR products. SNP typing was performed on amplified products with 5 μL of reaction mixture consisting of 2.5 μL of Premix Ex Taq (Probe qPCR) (Takara Bio Inc.), 0.2 μM of each primer, 0.1 μM of LNA probe set, 0.05 μL of ROX Reference Dye II, and 0.5 μL of template DNA using the 7500 Real-Time PCR System (Applied Biosystems). Primers and probes for SNP assay are also shown (Additional file 2). The probes for detecting wild type and mutant SNPs were labeled with HEX and 6-FAM (6-carboxyfluorescein) as reporters at 5′ end, respectively. All probes contained Iowa Black^®^ FQ (IBFQ) as a dark quencher at 3′ end.

To evaluate performance of the SNP assay system in all 6 genes, two *P. falciparum* strains, wild type (3D7) and a mutant-type (a strain from Thailand identified during preliminary experiments) were used as positive controls. Nucleotide sequence data are available in the GenBank™, EMBL, and DDBJ databases under the accession numbers: LC193525–LC193693.

### Statistical analysis

Kaplan–Meier product limit formula was used for the estimation of day 28-PCR-adjusted cure rates, which was the primary efficacy endpoint. The uncorrected and PCR-corrected Kaplan–Meier cumulative treatment success rates up to day 28 were calculated for all participants. Patients were censored when either lost to follow-up or withdrawn from the study. Either Fisher’s exact test or Pearson’s Chi square test (χ^2^) test were used for comparison of categorical variables, and Wilcoxon rank sum test was used for the continuous variables. *P* values <0.05 were considered statistically significant. R statistical software (version 3.2.0; R Foundation for Statistical Computing) was used for all the statistical analyses.

## Results

### Studied individuals

**Fig. 1:**
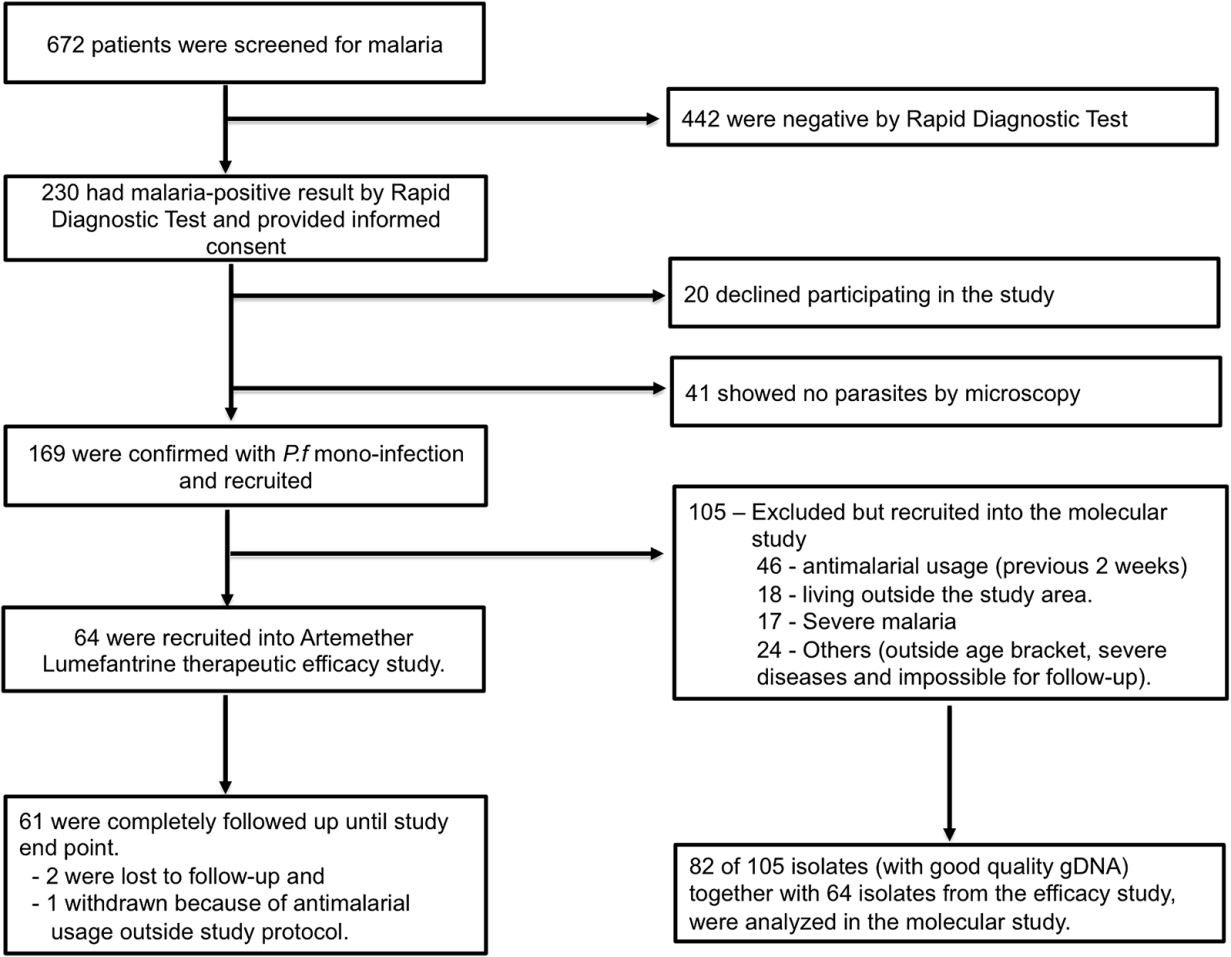
Study design of artemether–lumefantrine therapeutic efficacy study

A total of 672 patients were screened, out of which 169 microscopically confirmed *P. falciparum* mono-infection were recruited. Sixty-four of the 169 patients met the inclusion criteria for the AL therapeutic efficacy study (Fig. 1). The remaining 105 patients were excluded due to: anti-malarial-drug usage within two weeks (n = 46), living outside the study catchment area (n = 18), severe malaria (n = 17) and for other reasons (n = 24). Of the 64 enrolled individuals, two were lost to follow-up and one was withdrawn for taking anti-malarial drugs outside the study protocol. This resulted in 61 successfully followed-up cases (retention rate = 95.3%). Mean age and parasitaemia of the studied individuals were 3.3 years and 11,579 μL (parasite density = 0.26%), respectively (Table 1). Older children (≥5 years) tended to have higher parasitaemia as compared to the younger children <5 years though not statistically significant (*p* = 0.31). Other characteristics remained comparable between age groups.

**Table 1:** Characteristics of 61 individuals in artemether–lumefantrine efficacy study at enrollment

Characteristic	<5 years (n = 49)	≥5 years (n = 12)
Gender ratio (male/female)	32/17	5/7
Age (years)	2.7 ± 13.0	5.9 ± 18.8
Temperature (°C)	38.4 ± 1.3	38.5 ± 1.0
Geometric mean parasitaemia (No. of parasites/μL)	10,429.8 ± 10.4	17,746.4 ± 12.4
Haemoglobin (g/dL)	10.0 ± 1.9	10.8 ± 2.5
Gametocytes present (n)	4 (8.2%)	1 (8.3%)

± values are means and SD

### Artemether–lumefantrine treatment outcomes

Excellent early response to AL treatment was observed in both age groups (Table 2). Fever was cleared by day 2 in almost all patients. Only one (1.6%) child (4 years and 9 month-old male) showed microscopically residual parasites on day 3. His parasite density was 1.0% at enrollment, gradually decreased on days 1 (0.46%) and 2 (0.38%), and was still present by day 3 (0.22%, 9960 parasites/µL). The 3 days rescue regimen of dihydroartemisinin–piperaquine was, therefore, administered, and live parasites disappeared. However, pyknotic parasites persisted until day 7. Prevalence of parasite-positive individuals on day 3 as assessed by PCR was 22.9%, whereas it was 1.6% by microscopy. Individuals that were parasite positive by PCR (19,026.9 ± 11.3, n = 11) on day 3 had significantly (*p* = 0.0111) higher median parasitaemia at enrollment than the PCR negative group (9987.1 ± 10.5, n = 48). This suggests that parasite biomass before treatment may also be associated with treatment success and PCR parasite-positive outcome on day 3 (Additional file 3).

**Table 2:** Response to artemether–lumefantrine treatment

Characteristic	Age		Total (n = 61)
	<5 years (n = 49)	≥5 years (n = 12)	
Fever (≥37.5 °C) persistence [n (%)]			
Day 1	11 (22.4)	1 (8.3)	12 (19.7)
Day 2	3 (6.1)	0	3 (4.9)
Day 3	0	0	0
Parasite persistence			
Microscopy [n (%)]			
Day 1	40 (81.6)	11 (91.7)	51 (83.6)
Day 2	13 (26.5)	5 (4.2)	18 (29.5)
Day 3	1 (2.0)	0	1 (1.6)
PCR [n (%)]			
Day 1	45 (91.8)	11 (91.7)	56 (91.8)
Day 2	27 (55.1)	6 (50)	33 (54.1)
Day 3	11 (22.4)	3 (25)	14 (22.9)
Gametocyte persistence [n (%)]			
Day 1	4 (8.2)	1 (8.3)	5 (8.2)
Day 2	3 (6.1)	1 (8.3)	4 (6.6)
Day 3	3 (6.1)	1 (8.3)	4 (6.6)
28-day WHO treatment outcome [n (%)]			
Early treatment failure	0	0	0
Late clinical failure	2 (4.1)	1 (8.3)	3 (4.9)
Late parasitological failure	0	0	0
Adequate clinical and parasitological response	47 (96.0)	11 (91.7)	58 (95.1)
Cure rate [n (%)]			
PCR unadjusted	47 (96.0)	11 (91.7)	58 (95.2)
PCR adjusted	47 (96.0)	11 (91.7)	58 (95.2)

*PCR* polymerase chain reaction, *WHO* World Health Organization

Similar to the excellent early treatment response, cumulative-efficacy of AL was high with ACPR ratio of 95.2% (Table 2). Only three individuals showed LCF on day 28 and were later confirmed as reinfection by *msp1*, *msp2* and *glurp* genotyping (Additional file 4). These children were all male, aged; 11 months, 3 and 6 years with enrollment temperature of 36.8, 39.2 and 39.1 °C and initial parasitaemia of 1400 μL (0.03%), 99,200 μL (2.2%) and 96,080 μL (2.1%), respectively. Although all were parasite negative by day 2, they had malaria recurrence on day 28 with parasitaemia of 1840 μL (0.04%), 8460 μL (0.2%) and 32,120 μL (0.7%), respectively. The children were successfully treated with the rescue regimen. Gametocytes were observed in five individuals (8.2%) at enrolment, and in two cases, persisted until day 7 even after AL treatment.

### Drug-resistance related alleles at enrollment

In addition to 64 patients for the therapeutic efficacy study, 105 patients who did not meet inclusion criteria for the therapeutic efficacy study were recruited into the molecular epidemiological study for drug resistance. In total, 146 *P. falciparum* confirmed blood samples (82/105 with good quality gDNA + 64 from the therapeutic efficacy study) were used for genotyping of drug-resistance related alleles. Amino acid substitutions in Pfkelch13, which have been associated with artemisinin resistance in the Greater Mekong Sub region [11] were not detected; almost all (98.6%) carried wild-type allele and only A578S mutation was observed in two samples (1.4%) (Table 3). The six SNPs previously identified as background genetic changes for artemisinin resistance in Southeast Asia, were also genotyped [12]. All isolates harboured wild-type alleles in *fd*, *mdr2*, *arps10*, *pph* and *pibp*. Mutant alleles were only observed in *pfcrt* as mixed alleles (I356 + 356T) (n = 3). Regarding the other polymorphisms in *pfcrt*, K76T, which is known to be the responsible genetic change for chloroquine resistance, was observed in 29.2% of the isolates. In *pfmdr1*, wild-type alleles were nearly fixed at all known polymorphic positions other than 184; position 86 (98.0%), 1034 (98.6%), 1042 (100%) and 1246 (93.7%), respectively. At position 184, the prevalence of wild type alleles was high (80.4%), but as above, was significantly lower than at the other positions (*p* < 0.0001 by Pearson’s Chi square test).

**Table 3:** Prevalence of amino acid substitutions in the putative drug-resistance related genes in *Plasmodium falciparum* isolates collected before artemether–lumefantrine treatment

Gene	Amino acid position	Genotypes		
		Wild type (n, %)	Mutant (n, %)	Mixed (n, %)
*Pfkelch13*		141 (98.6)	2 (1.4) A578S	0
*fd*	D193Y	127 (100)	0	0
*mdr2*	T484I	126 (100)	0	0
*arps10*	V127M	127 (100)	0	0
*pph*	V1157	122 (100)	0	0
*pibp*	C1484F	127 (100)	0	0
*Pfcrt*	K76T	98 (68.1)	42 (29.2)	4 (2.7)
	I356T	122 (97.6)	0	3 (2.4)
*Pfmdr1*	N86Y	141 (98.0)	3 (2.1)	0
	Y184F	115 (80.4)	14 (9.8)	14 (9.8)
	S1034C	143 (98.6)	2 (1.4)	0
	N1042D	145 (100)	0	0
	D1246Y	134 (93.7)	9 (6.0)	0

### In vivo selection of drug-resistance related alleles after artemether–lumefantrine treatment

**Fig. 2:**
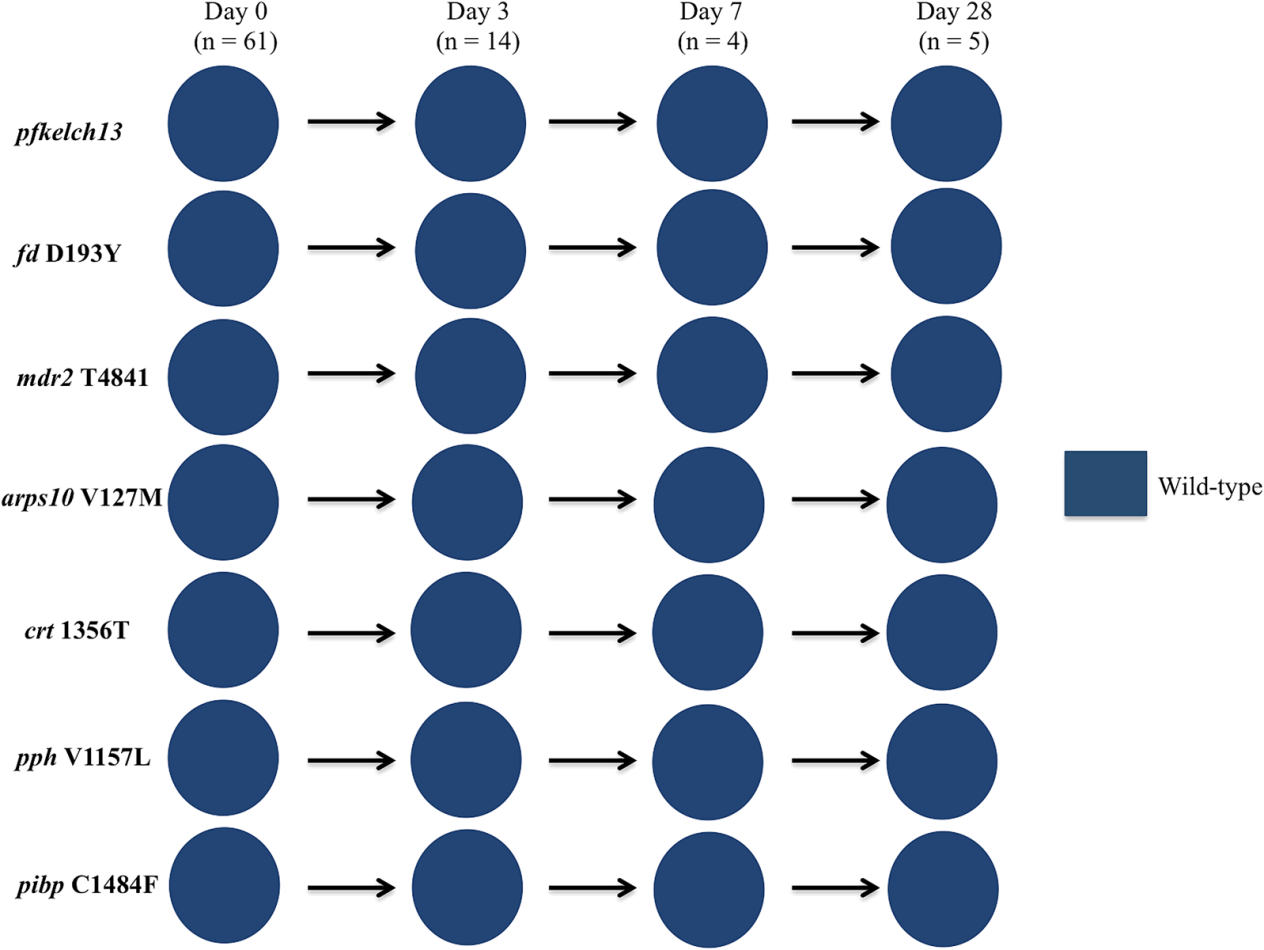
Allele prevalence in *Pfkelch13, fd, mdr2, arps10, crt, pph* and *pibp* among 61 isolates collected before and after artemether–lumefantrine treatment. Parasite genotypes were characterized at the time of presentation with malaria (day 0) and for infections detected within 28 days after treatment with artemether–lumefantrine (AL). *n values* represent the number of samples analysed on each day. Wild-type genotypes are indicated

**Fig. 3:**
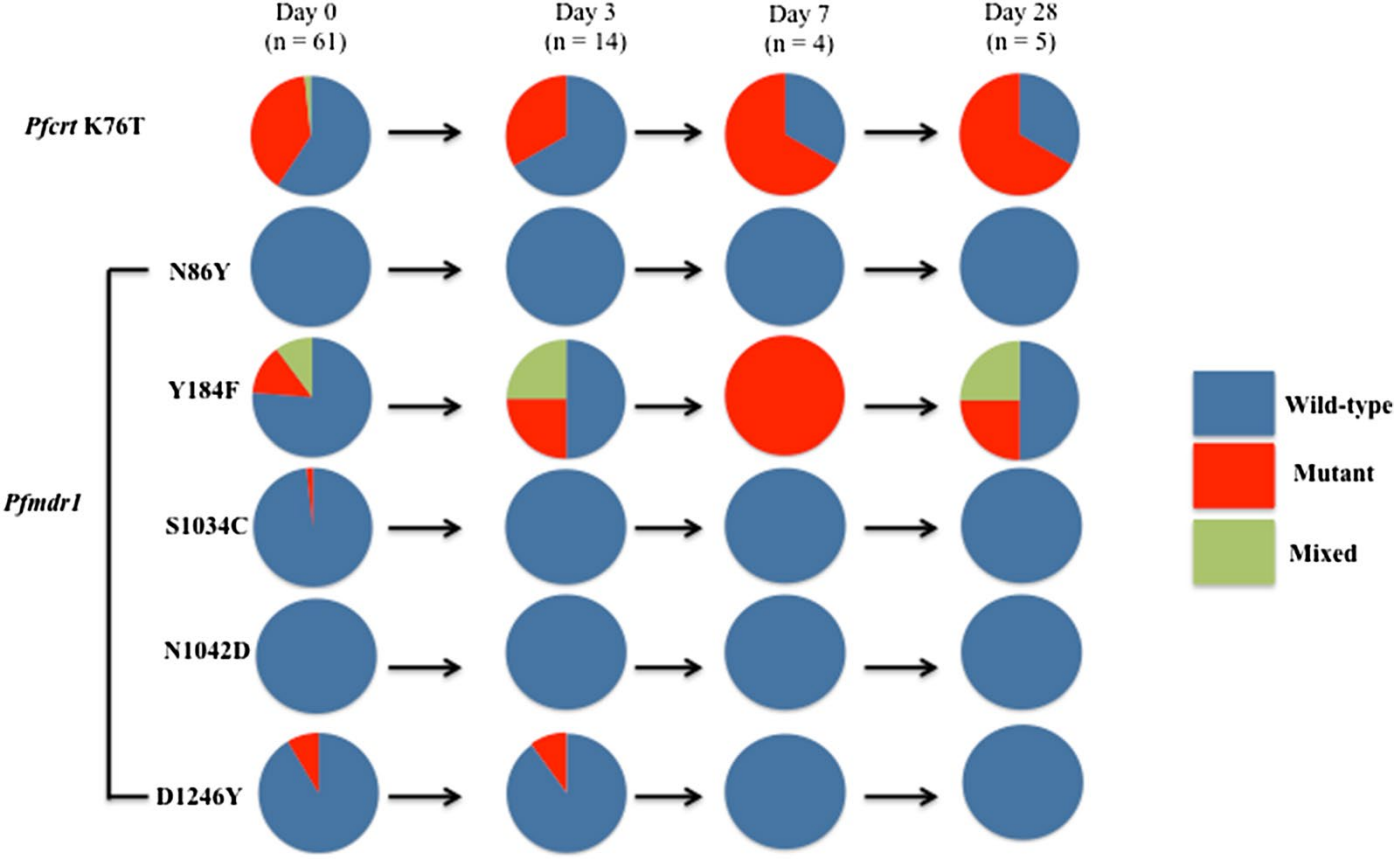
Allele prevalence in *pfcrt* K76T and *pfmdr1*among 61 isolates collected before and after artemether–lumefantrine treatment. Parasite genotypes were characterized at the time of presentation with malaria (day 0) and for infections detected within 28 days after treatment with artemether–lumefantrine (AL). *n values* represent the number of samples analysed on each day. Wild-type, mixed, and mutant genotypes are indicated

*Plasmodium falciparum* positivity after AL treatment was assessed by PCR in 61 individuals who were treated and successfully completed 28 days of follow-up. There were 23 PCR positive samples: 14 on day 3, 4 on day 7, and 5 on day 28. In *pfkelch13* and the six background genes for artemisinin resistance (genes encoding for ferredoxin, multiple resistance protein 2, apicoplast ribosomal protein S10, PfCRT protein, protein phosphatase and phosphoinositide-binding protein), all isolates carried wild-type alleles on day 3, 7 and 28 (Fig. 2). Amino acid position 76 in *pfcrt,* wild-type alleles were predominant at both day 0 (66.3%) and the follow-up period (50% on day 3, 66.7% on day 7, and 100% on day 28) (Additional file 5). In *pfmdr1*, wild-type alleles were nearly fixed at all polymorphic amino acid positions except for 184 on day 0. These alleles were completely fixed on the all follow-up days (day 7 and 28) as well. In contrast, position 184 remained polymorphic throughout the follow-up period without any trend of particular alleles except on day 7 where mutant alleles predominated (Fig. 3).

## Discussion

The present study revealed that both early and late responses to AL were still excellent in this study region of northern Uganda even after 8 years of its actual implementation as first-line treatment. Only one individual (1.6%) showed microscopically detectable parasites by day 3. Overall, similar excellent early response to AL treatment has been reported in other regions in Uganda [19–28]. According to WHO criteria [43] an endemic region showing ≥10% cases with detectable parasites on day 3 after ACT treatment is regarded as an area with suspected artemisinin resistance. The Worldwide Antimalarial Resistance Network (WWARN) proposes a more sensitive benchmark of 5% threshold for sub-Saharan Africa because of higher levels of herd immunity to malaria in the region [44]. In all cases, however, the prevalence of day 3 parasite positive individuals in the present study was less than the benchmarks for artemisinin-resistance.

PCR-confirmed parasite positivity after AL treatment was much higher than microscopically confirmed positivity; 91.8% on day 1, 54.1% on day 2, and 23% on day 3. These prevalences were similar to two previous studies that molecularly assessed parasite positivity in Kenya and Tanzania [45, 46]. Beshir et al. [45] reported that PCR-confirmed parasite positivity on day 3 would be a good predictor for malaria recurrence. In this study, however, recurrence frequencies did not differ much between the PCR-confirmed parasite positive group (7.7%) and the PCR-confirmed parasite negative group (4.2%) on day 3.

Mechanisms of artemisinin resistance have been gradually uncovered albeit the overall picture has not been clarified. Enhanced stress response including activation of unfolded protein response and the PI3K/Pi3P/AKT pathway is thought to be the main mechanism for parasite survival in the presence of artemisinin [47, 48]. Pfkelch13 has been elucidated to be involved in these processes [49, 50]. In the present analysis, however, nearly all parasites harboured wild-type alleles in *pfkelch13* at enrollment. The only mutation observed in Pfkelch13 was A578S, which has been widely distributed in Africa [13, 14]. Computational modelling reported that A578S could potentially disrupt the normal function of the Pfkelch13 protein [51]. However, only one study has described a close link between A578S and prolonged parasite clearance after artemisinin treatment [52] and others reported no association [14, 18, 53]. Very recently, it has been described that introduction of A578S mutation into Dd2 did not change the in vitro artemisinin susceptibility determined by ring-stage survival assay [14]. This observation partially supports the idea that A578S is not an artemisinin resistance related mutation. However, acquirement of artemisinin resistance would be a consequence of multiple genetic changes. As observed in this study and others [12], genetic background was different between African and Southeast Asian parasites. Since Dd2 clone is derived from Indochina, similar transfection studies using African parasite in addition to further in vivo efficacy study and population genetic assessment would be required to determine the potential role of A578S mutation.

In vivo selection analysis revealed that *pfkelch13* mutation was not observed in the parasite positive samples on day 3, 7 and 28, consistent with the recent observations [18]. Also, no selection of putative six non-synonymous polymorphisms was observed, suggesting that these genetic changes would not be responsible for parasite persistence in the present study. In contrast, Pfcrt K76 and Pfmdr1 N86/D1246 were observed in all recurrent parasites. Prevalence of Pfmdr1 Y184F (33.3%) in the recurrent patients was higher than baseline (14%), although not statistically significant. These observations support the potential selection of Pfcrt K76 and Pfmdr1 N86/Y184F/D1246 after AL treatment [16, 17]. In vivo selection of these mutations would increase these allele prevalences in the parasite population. In fact, the analysis herein revealed much higher allele frequencies than previously reported [4]; 68 versus 0% in the Pfcrt K76, 98.0 versus 9.5% in Pfmdr1 N86 and 93.7 versus 16.9% in Pfmdr1 D1246.

One patient showed day 3 parasite positivity in the present study. Multiple factors are associated with delayed parasite clearance after AL treatment [54]. Quality-assured artemether–lumefantrine, Coartem®, was used and all treatment were administered at the study hospital using DOTS, suggesting that drug factors were very unlikely to have major contribution to the early treatment failure. High initial parasitaemia is considered to be the most contributing factor to parasite positivity on day 3 [44]. However, the baseline parasitaemia of this patient was only 0.85%, precluding this possibility. This individual suffered from sickle cell anaemia with a haemoglobin level of 7.8 g/dL. Splenic function to filter parasitized red blood cells is impaired in sickle cell anaemia patients [55]. Previous meta-analysis also reported that severe anaemia was associated with slow parasite clearance and indicative of a poor immune response [44, 56]. Additionally, infected parasites carried Pfmdr1 allele combination (N86 and D1246) that was reported to be associated with sub-microscopic residual parasites on day 3 [57]. Taken together, the host factors in addition to parasite innate resistant potential might have functioned in the observed impaired early response.

Role of the human immunodeficiency virus (HIV)-infection to malaria treatment efficacy with ACT remains to be elusive. Some studies showed a close association between HIV infection and lower ACT treatment outcome [58], but others negated this association [24, 59]. In this study, HIV infection was not determined, mainly because nearly all patients showed excellent ACT efficacy, and thus, this would not be suitable for the association study. However, future studies are expected to clarify the potential role of HIV infection to ACT efficacy.

Unlike previous studies with malaria recurrence rates of 35–60% after artemether–lumefantrine treatment in Uganda [20–24, 26], much lower rates (4.9%) were observed in the study area. This can be explained by considerably fewer cases of reinfection in this study. Moreover, this is most likely due to considerable decrease in malaria prevalence in the study site after the scaled-up indoor residual spraying program in the mid-northern region [32].

## Conclusion

This study demonstrated that AL treatment remains of high efficacy for the treatment of *P. falciparum* malaria after 8 years of use in a region of high malaria transmission in Uganda. No clear evidence was obtained for the selection of mutant alleles in *pfkelch13* and the six-background genes, all of which have been reported to be associated with artemisinin resistance in Southeast Asia. Close monitoring of AL efficacy is necessary as we seek to understand the influence of anti-malarial treatments and parasite persistence, malaria transmission setting and different genetic background of parasites and host interactions in the mutational process. This work was done where very low early treatment failure and recurrence as well as scarcity of mutant alleles in *pfkelch13* and the six-background genes were observed; if the circumstances change, repeated in vivo selection analysis is recommended.

## Additional files

**Additional file 1.** Eligibility criteria for recruitment into the therapeutic efficacy study and the molecular study.

**Additional file 2.** List of primers and probes for multiplex and qPCR for SNP typing in *Plasmodium falciparum*.

**Additional file 3.** Parasitaemia at enrollment and Day 3 parasite positivity determined by PCR.

**Additional file 4.** Recrudescence versus re-infection test results of recurrent malaria infections.

**Additional file 5.** Allele prevalence in *pfcrt* K76T and *pfmdr1*among 103 isolates collected before and after artemether–lumefantrine treatment.

## References

[1] WHO. World Malaria Report 2014. Geneva: World Health Organization; 2014. www.who.int/malaria/…/world_malaria_report_2014/wmr-2014-no-profiles.pdf. Accessed 4 Jan 2016.

[2] Eastman RT, Fidock DA (2009). Artemisinin-based combination therapies: a vital tool in efforts to eliminate malaria. Nat Rev Microbiol.

[3] Noedl H, Socheat D, Satimai W (2009). Artemisinin-resistant malaria in Asia. N Engl J Med.

[4] Ashley EA, Dhorda M, Fairhurst RM, Amaratunga C, Lim P, Suon S (2014). Spread of artemisinin resistance in *Plasmodium falciparum* malaria. N Engl J Med.

[5] Word Health Organization Global Malaria Programme. Status report on artemisinin resistance, September 2014. http://www.who.int/malaria/publications/atoz/status_rep_artemisinin_resistance_sep2014.pdf. Accessed 16 Nov 2015.

[6] Four Artemisinin-Based Combinations (4ABC) Study Group (2011). A head-to-head comparison of four artemisinin-based combinations for treating uncomplicated malaria in African children: a randomized trial. PLoS Med.

[7] Roper C, Pearce R, Nair S, Sharp B, Nosten F, Anderson T (2004). Intercontinental spread of pyrimethamine-resistant malaria. Science.

[8] Mita T, Venkatesan M, Ohashi J, Culleton R, Takahashi N, Tsukahara T (2011). Limited geographical origin and global spread of sulfadoxine-resistant dhps alleles in *Plasmodium falciparum* populations. J Infect Dis.

[9] Mita T, Tanabe K, Takahashi N, Culleton R, Ndounga M, Dzodzomenyo M (2009). Indigenous evolution of *Plasmodium falciparum* pyrimethamine resistance multiple times in Africa. J Antimicrob Chemother.

[10] Mita T, Tanabe K, Kita K (2009). Spread and evolution of *Plasmodium falciparum* drug resistance. Parasitol Int.

[11] Ariey F, Witkowski B, Amaratunga C, Beghain J, Langlois AC, Khim N (2014). A molecular marker of artemisinin-resistant *Plasmodium falciparum* malaria. Nature.

[12] Miotto O, Amato R, Ashley EA, MacInnis B, Almagro-Garcia J, Amaratunga C (2015). Genetic architecture of artemisinin-resistant *Plasmodium falciparum*. Nat Genet.

[13] MalariaGen. *P. falciparum* Community Project data (beta release). Oxford: MalariaGen, 2015. https://elifesciences.org/content/5/e08714. Accessed 18 Aug 2016.

[14] Ménard D, Khim N, Beghain J, Adegnika AA, Shafiul-Alam M, Amodu O (2016). A worldwide map of *Plasmodium falciparum* K13-propeller polymorphisms. N Engl J Med.

[15] Conrad MD, LeClair N, Arinaitwe E, Wanzira H, Kakuru A, Bigira V (2014). Comparative impacts over 5 years of artemisinin-based combination therapies on *Plasmodium falciparum* polymorphisms that modulate drug sensitivity in Ugandan children. J Infect Dis.

[16] Sisowath C, Petersen I, Veiga MI, Mårtensson A, Premji Z, Björkman A (2009). In vivo selection of *Plasmodium falciparum* parasites carrying the chloroquine-susceptible pfcrt K76 allele after treatment with artemether–lumefantrine in Africa. J Infect Dis.

[17] Happi CT, Gbotosho GO, Folarin OA, Sowunmi A, Hudson T, O’Neil M (2009). Selection of *Plasmodium falciparum* multidrug resistance gene 1 alleles in asexual stages and gametocytes by artemether–lumefantrine in Nigerian children with uncomplicated falciparum malaria. Antimicrob Agents Chemother.

[18] Muwanguzi J, Henriques G, Sawa P, Bousema T, Sutherland CJ, Beshir KB (2016). Lack of K13 mutations in *Plasmodium falciparum* persisting after artemisinin combination therapy treatment of Kenyan children. Malar J..

[19] Yeka A, Kigozi R, Conrad MD, Lugemwa M, Okui P, Katureebe C (2016). Artesunate/amodiaquine versus artemether/lumefantrine for the treatment of uncomplicated malaria in Uganda: a randomized trial. J Infect Dis.

[20] Yeka A, Lameyre V, Afizi K, Fredrick M, Lukwago R, Kamya MR (2014). Efficacy and safety of fixed-dose artesunate-amodiaquine vs. artemether–lumefantrine for repeated treatment of uncomplicated malaria in Ugandan children. PLoS ONE.

[21] Yeka A, Tibenderana J, Achan J, D’Alessandro U, Talisuna AO (2013). Efficacy of quinine, artemether–lumefantrine and dihydroartemisinin–piperaquine as rescue treatment for uncomplicated malaria in Ugandan children. PLoS ONE.

[22] Muhindo MK, Kakuru A, Jagannathan P, Talisuna A, Osilo E, Orukan F (2014). Early parasite clearance following artemisinin-based combination therapy among Ugandan children with uncomplicated *Plasmodium falciparum* malaria. Malar J..

[23] Kapisi J, Bigira V, Clark T, Kinara S, Mwangwa F, Achan J (2015). Efficacy and safety of artemether–lumefantrine for the treatment of uncomplicated malaria in the setting of three different chemopreventive regimens. Malar J..

[24] Arinaitwe E, Sandison TG, Wanzira H, Kakuru A, Homsy J, Kalamya J (2009). Artemether–lumefantrine versus dihydroartemisinin–piperaquine for falciparum malaria: a longitudinal, randomized trial in young Ugandan children. Clin Infect Dis.

[25] Kamya MR, Yeka A, Bukirwa H, Lugemwa M, Rwakimari JB, Staedke SG (2007). Artemether–lumefantrine versus dihydroartemisinin–piperaquine for treatment of malaria: a randomized trial. PLoS Clin Trials..

[26] Bukirwa H, Yeka A, Kamya MR, Talisuna A, Banek K, Bakyaita N (2006). Artemisinin combination therapies for treatment of uncomplicated malaria in Uganda. PLoS Clin Trials..

[27] Yeka A, Dorsey G, Kamya MR, Talisuna A, Lugemwa M, Rwakimari JB (2008). Artemether–lumefantrine versus dihydroartemisinin–piperaquine for treating uncomplicated malaria: a randomized trial to guide policy in Uganda. PLoS ONE.

[28] Dorsey G, Staedke S, Clark TD, Njama-Meya D, Nzarubara B, Maiteki-Sebuguzi C (2007). Combination therapy for uncomplicated falciparum malaria in Ugandan children: a randomized trial. JAMA.

[29] McNamara DT, Kasehagen LJ, Grimberg BT, Cole-Tobian J, Collins WE, Zimmerman PA (2006). Diagnosing infection levels of four human malaria parasite species by a polymerase chain reaction/ligase detection reaction fluorescent microsphere-based assay. Am J Trop Med Hyg.

[30] Uganda Bureau of Statistics. Uganda Malaria Indicator Survey 2009. https://dhsprogram.com/pubs/pdf/MIS6/MIS6.pdf. Accessed 8 Jan 2016.

[31] Okello PE, Van Bortel W, Byaruhanga AM, Correwyn A, Roelants P, Talisuna A (2006). Variation in malaria transmission intensity in seven sites throughout Uganda. Am J Trop Med Hyg.

[32] Uganda Bureau of Statistics (UBOS) and ICF International 2015. Uganda Malaria Indicator Survey 2014–2015. https://dhsprogram.com/pubs/pdf/MIS21/MIS21.pdf. Accessed 8 Jan 2016.

[33] World Health Organization. Methods for surveillance of antimalarial drug efficacy. http://apps.who.int/iris/bitstream/10665/44048/1/9789241597531_eng.pdf. Accessed 10 Jan 2016.

[34] Sakihama N, Mitamura T, Kaneko A, Horii T, Tanabe K (2001). Long PCR amplification of *Plasmodium falciparum* DNA extracted from filter paper blots. Exp Parasitol.

[35] Rubio JM, Benito A, Roche J, Berzosa PJ, Garcia ML, Mico M (1999). Semi-nested, multiplex polymerase chain reaction for detection of human malaria parasites and evidence of *Plasmodium vivax* infection in Equatorial Guinea. Am J Trop Med Hyg.

[36] Tanabe K, Sakihama N, Kaneko O, Saito A, Kimura M (1999). A PCR Method for molecular epidemiology of *Plasmodium falciparum Msp*-*1*. Tokai J Exp Clin Med.

[37] Worldwide Antimalarial Resistance Network-Tools and resources/procedures. http://www.wwarn.org/tools-resources/procedures. Accessed 8 Dec 2016.

[38] Imagej.nih.gov. https://imagej.nih.gov/ij. Accessed 9 Dec 2016.

[39] Duraisingh MT, Jones P, Sambou I, von Seidlein L, Pinder M, Warhurst DC (2000). The tyrosine-86 allele of the pfmdr1 gene of *Plasmodium falciparum* is associated with increased sensitivity to the anti-malarials mefloquine and artemisinin. Mol Biochem Parasitol.

[40] Takahashi N, Tanabe K, Tsukahara T, Dzodzomenyo M, Dysoley L, Khamlome B (2012). Large-scale survey for novel genotypes of *Plasmodium falciparum* chloroquine-resistance gene pfcrt. Malar J..

[41] Tamura K, Stecher G, Peterson D, Filipski A, Kumar S (2013). MEGA6: molecular evolutionary genetics analysis version 6.0. Mol Biol Evol.

[42] PlasmoDB database. http://plasmodb.org/plasmo/app/record/gene/PF3D7_1343700. Accessed 7 Jan 2015.

[43] WHO. Global plan for artemisinin resistance containment (GPARC). Geneva: World Health Organization. http://www.who.int/malaria/publications/atoz/artemisinin_resistance_containment_2011.pdf. Accessed 7 Jan 2016.

[44] WWARN Artemisinin based Combination Therapy (ACT) Africa Baseline Study Group (2015). Clinical determinants of early parasitological response to ACTs in African patients with uncomplicated falciparum malaria: a literature review and meta-analysis of individual patient data. BMC Med.

[45] Beshir KB, Sutherland CJ, Sawa P, Drakeley CJ, Okell L, Mweresa CK (2013). Residual *Plasmodium falciparum* parasitemia in Kenyan children after artemisinin-combination therapy is associated with increased transmission to mosquitoes and parasite recurrence. J Infect Dis.

[46] Carlsson AM, Ngasala BE, Dahlström S, Membi C, Veiga IM, Rombo L (2011). *Plasmodium falciparum* population dynamics during the early phase of anti-malarial drug treatment in Tanzanian children with acute uncomplicated malaria. Malar J..

[47] Mita T, Tachibana SI, Hashimoto M, Hirai M (2016). *Plasmodium falciparum* kelch 13: a potential molecular marker for tackling artemisinin-resistant malaria parasites. Expert Rev Anti Infect Ther.

[48] Paloque L, Ramadani AP, Mercereau-Puijalon O, Augereau JM, Benoit-Vical F (2016). *Plasmodium falciparum*: multifaceted resistance to artemisinins. Malar J..

[49] Mok S, Imwong M, Mackinnon MJ, Sim J, Ramadoss R, Yi P (2011). Artemisinin resistance in *Plasmodium falciparum* is associated with an altered temporal pattern of transcription. BMC Genom.

[50] Dogovski C, Xie SC, Burgio G, Bridgford J, Mok S, McCaw JM (2015). Targeting the cell stress response of *Plasmodium falciparum* to overcome artemisinin resistance. PLoS Biol.

[51] Mohon AN, Alam MS, Bayih AG, Folefoc A, Shahinas D, Haque R (2014). Mutations in *Plasmodium falciparum* K13 propeller gene from Bangladesh (2009–2013). Malar J..

[52] Hawkes M, Conroy AL, Opoka RO, Namasopo S, Zhong K, Liles WC (2015). Slow clearance of Plasmodium falciparum in severe pediatric malaria, Uganda, 2011–2013. Emerg Infect Dis.

[53] Ouattara A, Kone A, Adams M, Fofana B, Maiga AW, Hampton S (2015). Polymorphisms in the *K13*-*propeller* gene in artemisinin-susceptible *Plasmodium falciparum* parasites from Bougoula-Hameau and Bandiagara, Mali. Am J Trop Med Hyg..

[54] Worldwide Antimalarial Resistance Network (WWARN) AL Dose Impact Study Group (2015). The effect of dose on the antimalarial efficacy of artemether–lumefantrine: a systematic review and pooled analysis of individual patient data. Lancet Infect Dis..

[55] Adeloye A, Luzzatto L, Edington GM (1971). Severe malarial infection in a patient with sickle-cell anaemia. BMJ.

[56] Price RN, Simpson JA, Nosten FR, Luxemburger CH, Hkirjaroen LI, ter Kuile FE (2001). Factors contributing to anemia after uncomplicated falciparum malaria. Am J Trop Med Hyg.

[57] Henriques G, Hallett RL, Beshir KB, Gadalla NB, Johnson RE, Burrow R (2014). Directional selection at the pfmdr1, pfcrt, pfubp1, and pfap2mu loci of *Plasmodium falciparum* in Kenyan children treated with ACT. J Infect Dis.

[58] Birku Y, Mekonnen E, Björkman A, Wolday D (2002). Delayed clearance of *Plasmodium falciparum* in patients with human immunodeficiency virus co-infection treated with artemisinin. Ethiop Med J.

[59] Kakuru A, Achan J, Muhindo MK, Ikilezi G, Arinaitwe E, Mwangwa F (2014). Artemisinin-based combination therapies are efficacious and safe for treatment of uncomplicated malaria in HIV-infected Ugandan children. Clin Infect Dis.

