# Supplementary material for: Absence of in vivo selection for K13 mutations after artemether–lumefantrine treatment in Uganda
Source: Malar J. 2017 Jan 9;16:23. doi: 10.1186/s12936-016-1663-1 (PMC5223472; doi:10.1186/s12936-016-1663-1)
Supplement: Supplementary file 1 — Additional file 1. Eligibility criteria for recruitment into the therapeutic efficacy study and the molecular study. [file 12936_2016_1663_MOESM1_ESM.docx]

**Additional file 1: Eligibility Criteria for recruitment into the therapeutic efficacy study and Molecular study**

|  | **Therapeutic Efficacy Study** |
| --- | --- |
| *Inclusion criteria* | 1. Children aged 6 months to 10 years |
|  | 1. Fever (axillary temperature ≥ 37.5^o^C) or history of fever within the previous 24 hours. |
|  | 1. Body weight ≥ 5kg |
|  | 1. Uncomplicated malaria with asexual *P.falciparum* mono infection with parasitaemia ≤ 200,000/μL. |
|  | 1. Hemoglobin concentration ≥ 5g/dL |
|  | 1. No history of hypersensitivity reactions or contradictions to the study drug. |
|  | 1. No evidence of concomitant febrile illness |
|  | 1. Provision of informed consent by a parent or guardian and assent for children ≥ 7 years and agreement to complete 28 days of follow-up. |
|  | 1. No regular medication, which could interfere with antimalarial drug, *e.g*. prophylaxis with cotrimoxazole for the prevention of *Pneumocystis carinii* pneumonia in children born to HIV positive women. |
|  | 1. Absence of severe malnutrition (defined as a child whose growth standard is below –3 z-score, with symmetrical oedema involving at least the feet or mid-upper arm circumference < 110 mm). |
| *Exclusion Criteria* | 1. Concomitant severe disease (cardiac, renal, hepatic diseases) or co-infection with other malaria species, which would place the subject at undue risk or interfere with the results of the study. |
|  | 1. Danger signs or evidence of severe malaria defined as:   - Unarousable coma (if after convulsion, > 30 min)  - Recent convulsions (1 to 2 within 24 hours)  - Altered consciousness (confusion, delirium, psychosis, coma)  - Lethargy  - Severe anaemia (Hb < 5.0 g/dL)  - Respiratory distress (breathing difficulties/labored breathing at rest)  - Vomiting  - Prostration |
|  | 1. Antimalarial drug usage within 2 weeks prior to enrollment into the study. |
|  | 1. Presence of other conditions that in the opinion of the Investigator would jeopardize the safety or rights of a child in the study or would render the child unable to comply with the follow-up. |
|  | **Molecular Study** |
| *Inclusion criteria* | 1. Aged ≥ 6 months |
|  | 2. Uncomplicated malaria with asexual *P.falciparum* mono infection of any density. |
|  | 3. Provision of informed consent by a parent/guardian and assent for children ≥ 7 years. |
| *Exclusion Criteria* | None |
