# Supplementary material for: Absence of in vivo selection for K13 mutations after artemether–lumefantrine treatment in Uganda
Source: Malar J. 2017 Jan 9;16:23. doi: 10.1186/s12936-016-1663-1 (PMC5223472; doi:10.1186/s12936-016-1663-1)
Supplement: Supplementary file 2 — Additional file 2. List of primers and probes for multiplex and qPCR for SNP typing in Plasmodium falciparum. [file 12936_2016_1663_MOESM2_ESM.docx]

**Additional file 2: List of primers and probes for multiplex and qPCR for SNP typing in *P. falciparum***

| Primer Name | Full name of target genes | PlasmoDB ID | Type | Sequence (5′ - 3′) | # mer | Tm (^o^C) |
| --- | --- | --- | --- | --- | --- | --- |
| PfARPS10-F4  PfARPS10-R7 | Apicoplast ribosomal protein S10 precursor  Apicoplast ribosomal protein S10 precursor | PF3D7_1460900.1  PF3D7_1460900.1 | Multiplex PCR  Multiplex PCR | ATTGTAGCAGGCCCAATTCCCCAAAAGACA  CAGAATTTTTTATAGGAACAGATGAGTTCA | 30  30 | 77.1  64.9 |
| PfFD-F1 | Ferredoxin | PF3D7_1318100 | Multiplex PCR | TTGAATTTCCTAAGTAATAATCAGCTAGCT | 30 | 63.2 |
| PfFD-R1 | Ferredoxin | PF3D7_1318100 | Multiplex PCR | TATTAGGAAATTTTATCATTCCCCATTTCA | 30 | 66.9 |
| PfMDR2-F1 | Multidrug resistance protein 2+ (heavy metal transport family) | PF3D7_1447900 | Multiplex PCR | TGATGAAAATAGTGGCTTATTAGGAACAGA | 30 | 66.9 |
| PfMDR2-R1 | Multidrug resistance protein 2+ (heavy metal transport family) | PF3D7_1447900 | Multiplex PCR | ACCTGTATGACCTACAAGAGCACATGTTGT | 30 | 69.7 |
| PfPIBP-F2 | Phosphoinositide-binding protein | PF3D7_0720700 | Multiplex PCR | TACTTGAGAAGAATGTCATGAAAGA | 25 | 60.7 |
| PfPIBP-R1 | Phosphoinositide-binding protein | PF3D7_0720700 | Multiplex PCR | TGTTAGACATATCGTACATATCATACGCGT | 30 | 66.5 |
| PfCRT-F2 | Chloroquine resistance transporter | PF3D7_0709000 | Multiplex PCR | GAAAACCTTCGCATTGTTTTCCTTCT | 26 | 68.6 |
| PfCRT-R2 | Chloroquine resistance transporter | PF3D7_0709000 | Multiplex PCR | ATGATACGTTGTACCATCATAAACA | 25 | 60.8 |
| PfPPH-F3 | Protein phosphatase | PF3D7_1012700 | Multiplex PCR | GAAAAGTCAAGAGTCCTCTCAAGTGATTGA | 30 | 69.1 |
| PfPPH-R2 | Protein phosphatase | PF3D7_1012700 | Multiplex PCR | TCGATATTGAGTCAATATCGTACGCACAAC | 30 | 70.3 |
| PfARPS10-Fq | Apicoplast ribosomal protein S10 precursor | PF3D7_1460900.1 | qPCR | TTCGAATTGCTCTCTACTGTC | 21 | 59.7 |
| PfARPS10-Rq | Apicoplast ribosomal protein S10 precursor | PF3D7_1460900.1 | qPCR | AAAGACAATAAGAAAGAGGTAACATA | 26 | 59.4 |
| PfARPS10-probe (WT) | Apicoplast ribosomal protein S10 precursor | PF3D7_1460900.1 | Probe qPCR | TTAT+CC+A+C+A+TGGGG | 19 | 63.0 |
| PfARPS10-probe (Mut) | Apicoplast ribosomal protein S10 precursor | PF3D7_1460900.1 | Probe qPCR | A+TCCA+T+A+T+GGGG | 17 | 62.9 |
| PfFD-Fq | Ferredoxin | PF3D7_1318100 | qPCR | GATTGAAACGCACAAGGAAGAC | 22 | 62.1 |
| PfFD-Rq | Ferredoxin | PF3D7_1318100 | qPCR | CCCATTTCAATCATATCCATACCAAA | 26 | 62.0 |
| PfFD-probe (WT) | Ferredoxin | PF3D7_1318100 | Probe qPCR | AACTACA+C+G+ACATGTAAT | 21 | 61.8 |
| PfFD-probe (Mut) | Ferredoxin | PF3D7_1318100 | Probe qPCR | AACTA+CA+C+T+A+CATGTA | 21 | 62.4 |
| PfMDR2-Fq | Multidrug resistance protein 2+ (heavy metal transport family) | PF3D7_1447900 | qPCR | TAGTTGATAGAGGTACCGAGAG | 22 | 59.8 |
| PfMDR2-Rq | Multidrug resistance protein 2+ (heavy metal transport family) | PF3D7_1447900 | qPCR | ATAAAGTTAAACCTATAAATAATACACTACC | 31 | 59.7 |
| PfMDR2-probe (WT) | Multidrug resistance protein 2+ (heavy metal transport family) | PF3D7_1447900 | Probe qPCR | CCGGC+A+A+C+A+A+TAGAA | 21 | 63.5 |
| PfMDR2-probe (Mut) | Multidrug resistance protein 2+ (heavy metal transport family) | PF3D7_1447900 | Probe qPCR | CCGG+CA+A+T+A+A+TAGA | 20 | 61.7 |
| PfPIBP-Fq | Phosphoinositide-binding protein | PF3D7_0720700 | qPCR | TTGTTCATGTAAATGTTTGTAATTATAGGA | 30 | 61.6 |
| PfPIBP-Rq | Phosphoinositide-binding protein | PF3D7_0720700 | qPCR | TTCATATTCTGTCGAGGATAACAATTC | 27 | 61.7 |
| PfPIBP-probe (WT) | Phosphoinositide-binding protein | PF3D7_0720700 | Probe qPCR | CTA+CAAT+GAAAAAA+T+G+TAT+TAA | 28 | 64.9 |
| PfPIBP-probe (Mut) | Phosphoinositide-binding protein | PF3D7_0720700 | Probe qPCR | C+CTA+CAA+TGAAAAAA+T+T+TATTAA | 29 | 64.1 |
| PfCRT-Fq | Chloroquine resistance transporter | PF3D7_0709000 | qPCR | ACCATGACATATACTATTGTTAGTTGTA | 28 | 61.0 |
| PfCRT-Rq | Chloroquine resistance transporter | PF3D7_0709000 | qPCR | ATGATACGTTGTACCATCATAAACA | 25 | 61.0 |
| PfCRT-probe (WT) | Chloroquine resistance transporter | PF3D7_0709000 | Probe qPCR | A+G+CA+A+T+A+GCAAT | 18 | 61.1 |
| PfCRT-probe (Mut) | Chloroquine resistance transporter | PF3D7_0709000 | Probe qPCR | AGCA+A+C+A+GC+AAT | 17 | 60.8 |
| PfPPH-F1q-1 | Protein phosphatase | PF3D7_1012700 | qPCR | ACATATATGGATGATTATATGGATGGTAAA | 30 | 61.6 |
| PfPPH-R1q | Protein phosphatase | PF3D7_1012700 | qPCR | GTATTATTATTGACTTCGTTCACATGTTT | 29 | 61.8 |
| PfPPH-probe (WT) | Protein phosphatase | PF3D7_1012700 | Probe qPCR | TATT+CAAA+T+GT+CAAA+CA+TC | 25 | 66.1 |
| PfPPH-probe (Mut) | Protein phosphatase | PF3D7_1012700 | Probe qPCR | TATT+CAAA+T+C+TCAAA+CA+TCA | 26 | 67.0 |
