# Supplementary figures and images for: Absence of in vivo selection for K13 mutations after artemether–lumefantrine treatment in Uganda
Source: Malar J. 2017 Jan 9;16:23. doi: 10.1186/s12936-016-1663-1 (PMC5223472; doi:10.1186/s12936-016-1663-1)

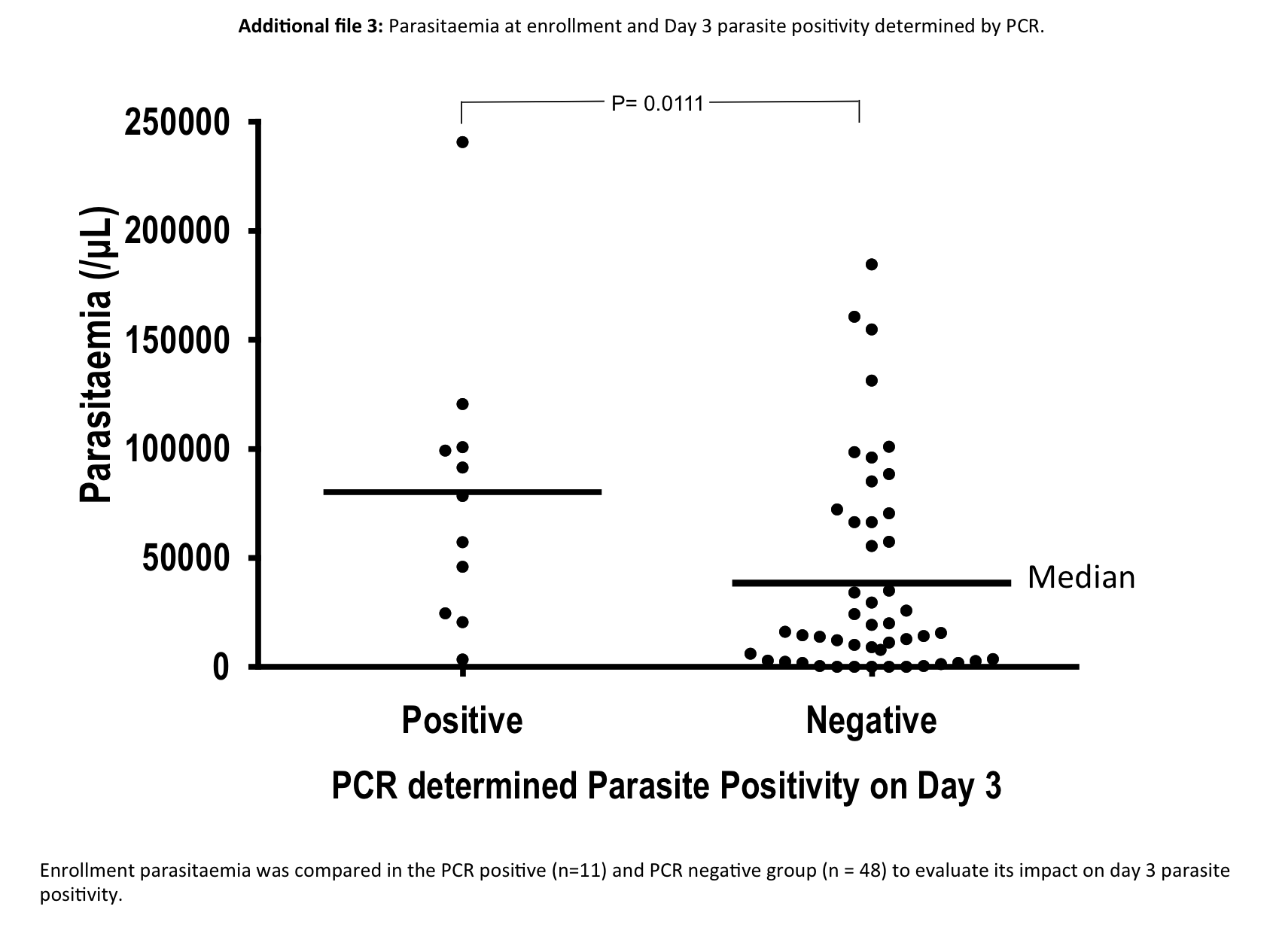

Supplement: Supplementary file 3 — Additional file 3. Parasitaemia at enrollment and Day 3 parasite positivity determined by PCR. [file 12936_2016_1663_MOESM3_ESM.tif]

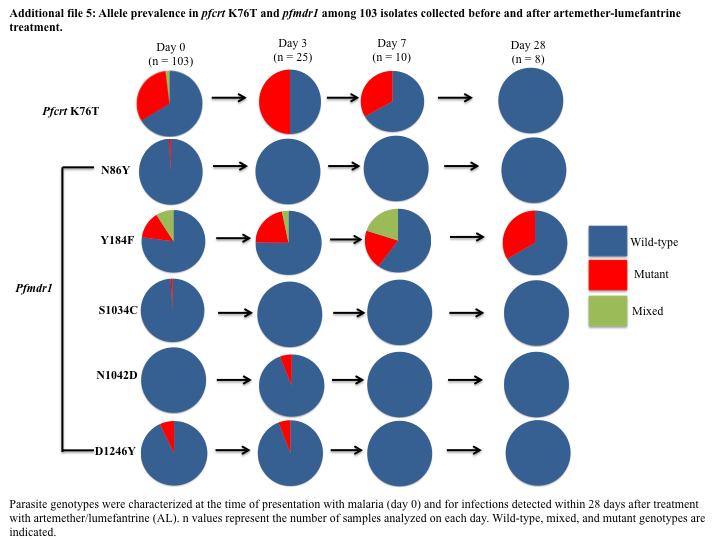

Supplement: Supplementary file 5 — Additional file 5. Allele prevalence in pfcrt K76T and pfmdr1among 103 isolates collected before and after artemether–lumefantrine treatment. [file 12936_2016_1663_MOESM5_ESM.tiff]
