# Supplementary material for: Absence of in vivo selection for K13 mutations after artemether–lumefantrine treatment in Uganda
Source: Malar J. 2017 Jan 9;16:23. doi: 10.1186/s12936-016-1663-1 (PMC5223472; doi:10.1186/s12936-016-1663-1)
Supplement: Supplementary file 4 — Additional file 4. Recrudescence versus re-infection test results of recurrent malaria infections. [file 12936_2016_1663_MOESM4_ESM.docx]

**Additional file 4:** **Recrudescence versus re-infection test results of recurrent malaria infections**

|  | **Sample**  **ID** | PCR Product sizes (bp) | | | | | | | | | | **Results/Comment** |
| --- | --- | --- | --- | --- | --- | --- | --- | --- | --- | --- | --- | --- |
|  |  | ***msp1* (Block 2 to 6*)** | | | | | ***msp1* (Block 4a/4b*)** | | | ***msp2*** | ***glurp*** |  |
|  |  | K1 | 3D7 | FCR3 | MAD20 | Thai838 | 97S | K1 | MAD20 | **3D7** | **3D7** |  |
| 1 | 1405-A024_D0 |  | 1100 |  |  |  | 94 |  |  | 240 | 950 | Re-infection |
| 2 | 1405-A024_D28 |  | 1100 |  |  |  | 94 |  |  | 320 | 900 |  |
|  |  |  |  |  |  |  |  |  |  |  |  |  |
| 3 | 1410-C036_D0 |  | 1100 |  |  | 1100 |  |  | 97 | 350 | 1000 | Re-infection |
| 4 | 1410-C036_D28 |  | - |  |  | 1100 |  |  | 97 | 290 | 900 |  |
|  |  |  |  |  |  |  |  |  |  |  |  |  |
| 5 | 1410-C088_D0 |  |  |  |  | 1100 | - |  | 97 | 260 | 810 | Re-infection |
| 6 | 1410-C088_D28 |  |  |  |  | 1100 | 94 |  | 97 | 250 | 1000 |  |

*For *msp1*, block sizes are after Kaneko O, Kimura M, Kawamoto F, Ferreira MU, Tanabe K. *Plasmodium falciparum*: allelic variation in the merozoite surface protein 1 gene in wild isolates from southern Vietnam. Exp Parasitol.1997;86:45-57.
